# Pre- or Post-Harvest Treatment with MeJA Improves Post-Harvest Storage of Lemon Fruit by Stimulating the Antioxidant System and Alleviating Chilling Injury

**DOI:** 10.3390/plants11212840

**Published:** 2022-10-25

**Authors:** Ling Liao, Sichen Li, Yunjie Li, Zehao Huang, Jiahao Li, Bo Xiong, Mingfei Zhang, Guochao Sun, Zhihui Wang

**Affiliations:** 1 College of Horticulture, Sichuan Agricultural University, Chengdu 611130, China; 2 Citrus Research Institute, Southwest University, Chongqing 400700, China; 3 Institute of Pomology and Olericulture, Sichuan Agricultural University, Chengdu 611130, China

**Keywords:** methyl jasmonate, lemon, fruit preservation, cold storage

## Abstract

Cold storage preserves lemon fruit quality; however, it can result in significant chilling injury (CI). The effects of pre- and post-harvest methyl jasmonate (MeJA) treatments at four concentrations (0, 0.1, 0.3, and 0.5 mM) on CI and sensory quality of lemons during 80 d of storage at 7–10 °C were investigated. Both pre- and post-harvest MeJA treatments reduced CI, weight loss (WL) and maintained higher firmness, total soluble solids (TSS), and total acidity (TA) than in the controls. Antioxidant enzyme activities decreased in the control fruit but increased in both pre- and post-harvest MeJA-treated fruit. In addition, phospholipase D (PLD) and lipoxygenase (LOX) activities and malondialdehyde (MDA) content were higher in the control than in the MeJA-treated fruit. Pre-harvest MeJA treatment generally preserved fruit better than post-harvest MeJA treatment, with the best results observed when MeJA was applied at 0.3 mM, which enhanced the antioxidant system of the lemon fruits, thus reducing the post-harvest incidence of chilling injury. These results have important implications for improved fruit quality post-harvest.

## 1. Introduction

Lemons (*Citrus limon* (L.) Burm. F.) rank as the third most important cultivated citrus and are popular for their favorable flavor and natural ingredients such as vitamins C and phenolic compounds [1]. Anyue county in Sichuan province grows 353 hm^2^ of ‘Eureka’ lemons, accounting for 80% of China’s lemon production. Cold storage is generally employed for retarding respiration and other metabolic processes in order to maintain the post-harvest fruit quality [2]. As a chilling-sensitive fruit, lemons are vulnerable to chilling injury (CI) below 10 °C [3], which manifests itself as rind staining, pitting, red blotches, scald, and watery breakdown on the flavedo, reducing fruit quality, shelf-life, and marketability [4].

CI is visible in the flavedo of citrus fruit and is triggered by the uncontrolled production of reactive oxygen species (ROS) [5]. Low temperatures can accelerate the production of ROS in lemons, and this hence leads to oxidative stress [6]. Due to the poisonous and detrimental effect that high concentrations of ROS have in cells, a tight control of ROS levels is indispensable for cells to avoid oxidative injury while not completely eliminating ROS [7]. Protection of cells from oxidative injury under chilling is thought to be a major mechanism of chilling resistance, and this resistance may be related to the level of antioxidant enzyme activity. Several enzymes in plant tissues are scavengers for ROS, including superoxide dismutase (SOD), catalase (CAT), and peroxidase (POD). These antioxidant enzymes can scavenge ROS, preventing the harmful effects of H_2_O_2_ in plant tissues [2]. Various methods can be used to reduce the occurrence of CI in citrus, including pre-storage techniques such as the application of salicylic acid (SA), methyl jasomonate (MeJA), fungicides, and wax, and post-harvest technologies, including intermittent warming (IW) and temperature conditioning (TC) [8].

MeJA is deemed as a modulator in many physiological activities in higher plants, for instance, flowering, defense responses, and senescence [9]. MeJA is classified as a “generally recognized as safe” (GRAS) substance by the U.S. Food and Drug Administration [10]. The pre- and post-harvest application of MeJA to horticultural crops has been widely studied [11]. For instance, pre-harvest MeJa treatment in plums [12] and pomegranates [13] showed a delay in fruit decay and better post-harvest quality; post-harvest MeJa treatment before storage at chilling temperature reduced CI in blood oranges [14], apple [15] and kiwifruit [16]. García-Pastor et al. (2020) [17] evaluated for the first time the effects of pre-harvest MeJA treatments, alone or in combination with post-harvest MeJA treatment, on reducing CI in pomegranates and the results showed that there were no significant differences between pre- and post-harvest MeJa treatments in their effects on reducing CI, maintaining membrane stability and bioactive compounds with antioxidant activity. Specifically in lemon fruit, Siboza et al. (2017) [6] showed that post-harvest treatments with MeJA and SA alone or in combination, decreased chilling injury damage during lemon storage at cold temperatures, Serna-Escolano et al. [18] suggested that pre-harvest MeJA treatments increase CAT, POD, and APX activities in lemons. Rehman et al. [19] reported that post-harvest treatment with 0.25 mM MeJA followed by 90 d cold storage and 10 d simulated shelf conditions were free from CI in ‘Midknight’ Valencia, irrespective of the cold storage temperature; although the mechanism involved in this effect has not been elucidated yet and deserves further research.

The objective of the present study was to evaluate the effects of pre-harvest MeJA treatments alone, and post-harvest MeJA treatment alone, on reducing CI in ‘Eureka’ lemons and its relationship with changes in antioxidant enzymes during storage. In addition, the fruits’ quality and their texture were also evaluated. These results provide theoretical support for a better understanding of lemon preservation with pre-harvest MeJA treatments and post-harvest MeJA treatment.

## 2. Materials and Methods

### 2.1. Plant Material

Commercial fields located in Anyue County (Sichuan, China) were used to perform the experiments in 2020 using lemons (*Citrus limon* L. cv. ‘Eureka’) grafted onto trifoliate oranges [*Poncirus trifoliata* (L.) Raf] rootstock, under standard cultural practices. The trees were planted at 3 × 4 m, with the ‘Eureka’ trees being 10 years old. Each experimental tree was fertilized with 5000 g organic fertilizer, 1200 g of nitrogen [CO(NH_2_)_2_; N ≥ 46.0%], 750 g of phosphorus [NH_4_H_2_PO_4_; P_2_O_5_ ≥ 44.0%], 800g of potassium [K_2_SO_4_; K_2_O ≥ 50.0%] per year and received standard irrigation, and plant protection.

### 2.2. Pre-Harvest MeJA Treatments

Field experiments were performed at random using four blocks of five trees for each treatment. ‘Eureka’ plants were randomly divided into four groups. The trees were treated when the lemons were in the color-change period (90 d after flowering) which was the time before bagging. Treatments were performed by applying 5 L of freshly prepared MeJA at 0.1, 0.3 or 0.5 mmol L^−1^, containing 0.5 mL L^−1^ Tween-20. The control trees were sprayed with distilled water containing 0.5 mL L^−1^ Tween-20. The fruits were harvested on 15th December (230 d after flowering). One hundred fruits (homogeneous in size and color) from each treatment were taken and transported immediately from the collection site to the laboratory (The fifth experimental building, Sichuan Agricultural University, Chengdu, China) and all fruit coverage with PE (for preventing the spread of decay). For each treatment the harvested fruits divided into five lots of twenty fruits and stored at 7–10 °C at a relative humidity of 85% to 90%. One lot of twenty fruits for each replicate and treatment was sampled at random after 0, 20, 40, 60 and 80 days of cold storage for analytical determinations.

### 2.3. Post-Harvest MeJA Treatment

Three hundred fruits without pre-harvest MeJA treatment and without physical injuries or apparent decay were randomly harvested. All the selected fruits were divided into three groups with a randomized method soon after they were transported to the laboratory, each consisting of 100 fruits. The fruits were dipped for five minutes in a solution of 0.1, 0.3, or 0.5 mM MeJA and 0.5 mL L^−1^ Tween-20 as a surfactant. For each treatment, the fruits were divided into five lots of twenty fruits, then all the fruits went through the process of air-drying, being packaged into plastic trays, covered with PE (wrap film), and stored at 7–10 °C at a relative humidity of 85% to 90%. One lot of twenty fruits for each replicate and treatment was sampled at random after 0, 20, 40, 60 and 80 days of cold storage for analytical determinations.

### 2.4. Phytochemical Parameters

Each individual fruit was weighed on day 0 and after each sampling date during storage, and weight loss rate (WL) was expressed as a percentage (%). The texture profile analysis (TPA) was carried out following Qiu et al. (2021) [20], with modifications, using a texture profile analyzer (TMS-Pilot, Ensoul Technology Ltd., Beijing, China). Springiness, firmness, gumminess, and chewiness were recorded (Table 1). For electrolyte leakage (EL) evaluation, lemon fruits from each treatment were randomly sampled and peel samples (2 g) were placed in a 25 mL beaker with 20 mL of distilled water and shaken at room temperature for 3 h. First electrolyte leakage (EC1) and second electrolyte leakage (EC2) were measured after 3 h of shaking and after autoclave at 121◦C for 20 min and cooling down at room temperature, respectively, using a DDS- II conductometer. EL (%) = EC1/EC2×100. The CI index was calculated visually using a scale of 0–4 based on the percentage of lemon fruit surface area exhibiting pitting and/or dark watery patches as follows: 0 = no symptoms; 1 = slight injury (1–25%); 2 = moderate injury (26–50%); 3 = severe injury (51–75%); and 4 = extremely severe injury (76–100%). CI index (between 0 and 4) = Σ(CI scale) × (number of fruit at the CI level)/(total number of fruits in the treatment).

### 2.5. Fruit Internal Quality

The juice of the 20 fruits from each replicate was then mixed and used to measure total soluble solids (TSS), titratable acidity (TA) and Vitamin C (V_C_) in duplicate in each sample. The TSS was measured using a hand-held refractometer (Atago Co. Ltd., Tokyo, Japan), and the TA was measured using 10 mL fruit juice diluted with distilled water (1:2) and titrated to pH 8.2 using 0.1 N NaOH. The Vc was measured using 2,6-dichlorophenol-indophenol-titration.

### 2.6. Activity of the Antioxidant Enzymes

The activity of phospholipase D (PLD) was determined on the basis of the method of Sirikesorn et al. [21]. One unit of PLD activity was defined as the amount of enzyme which converted substrate to 1 nmol p-nitrophenol per hour with the expression of U mg^−1^ pro. The activity of LOX was determined based on the method of Podazza et al. [22], one unit of LOX activity was defined as an increase of ∆OD_234_ by 0.1 within 1 min. Aliquots (0.5 g) of tissue samples were homogenized in 5 mL of phosphate buffer (0.1 M, pH 7.8) and centrifuged at 10,000× *g* for 20 min at 4 °C. The supernatant was used to measure SOD, POD, CAT, and APX activities and MDA contents. One unit (U) of SOD activity was calculated as the quantity of enzyme which causes 50% of nitroblue tetrazolium reduction per min, one enzyme unit (U) was defined as a 0.01 decrease in absorbance at 290 nm min^−1^ for APX, a 0.01 increase of absorbance at 470 nm min^−1^ for POD and, a 0.01 decrease of absorbance at 240 nm min^−1^ for CAT. The content of MDA was determined using thiobarbituric acid (TBA).

### 2.7. Statistical Analysis

The results obtained were expressed as the mean ± SE of three randomized replicates. Data were subjected to analysis of variance (ANOVA) and a multiple comparison test with the least significant differences (LSD) test being carried out to find significant differences (*p* < 0.05) among the treatments, using SPSS, version 22 (IBM Corp., Armonk, NY, USA). Principal component analysis (PCA) was performed using Origin 8.1 software (OriginLab Corporation, Northampton, MA, USA).

## 3. Results

### 3.1. Chilling Injury and Weight Loss during Cold Storage

**Figure 1:**
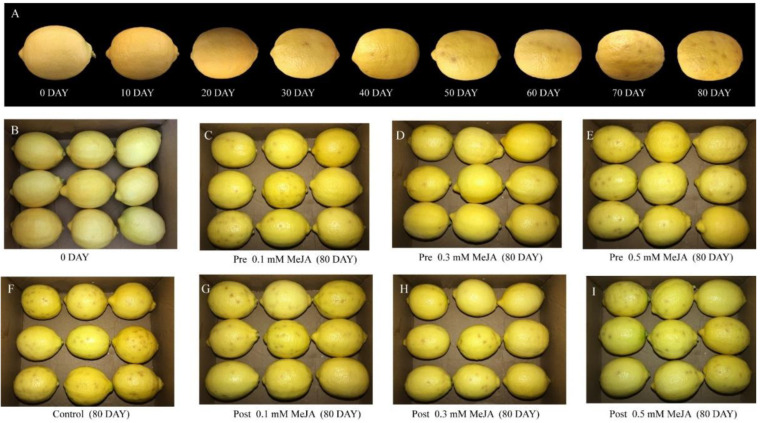
External appearance of ‘Eureka’ lemon during storage at 7–10 °C. (**A**) External appearance changes of control fruits after 0, 10, 20, 30, 40, 50, 60, 70 and 80 days of cold storage; (**B**) External appearance of all treated fruits in cold storage for 0 day; (**C**–**E**) External appearance of fruits treated with pre-harvest 0.1, 0.3, and 0.5 mM MeJA after 80 days of cold storage, respectively; (**F**) External appearance of control fruits after 80 days of cold storage; (**G**–**I**) External appearance of fruits treated with post-harvest 0.1, 0.3, and 0.5 mM MeJA after 80 days of cold storage, respectively.

**Figure 2:**
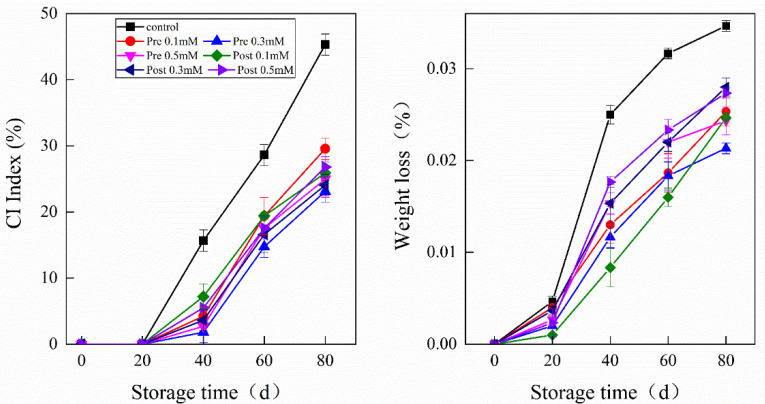
Effect of pre-harvest (Pre) and post-harvest (Post) MeJA treatments on weight loss in lemons at storage at 7−10 °C. Data are the mean ± SE of three replicates of five fruit.

External chilling injury (CI) symptoms were manifested by depression of the fruit surface, browning, rind staining and pitting (Figure 1A), after 20 days in storage; the severity increased with storage time. For both pre- and post-harvest MeJA-treated fruits, the increases in CI indexes were significantly lower (*p* < 0.05) than in the controls at the same storage period. However, differences between pre- and post-harvest treatments were not significant for CI of storage. Overall, all MeJA treatments lowered CI in a great sense, causing delays to the appearance of CI symptoms during the storage of lemon fruits at 7–10 °C. It was found that pre-harvest treatment with 0.3 mM MeJA significantly reduced the CI index, resulting in a CI of 23.13% on day 80 compared with 45.33% in the control fruit, while pre-harvest treatment with 0.5 mM MeJA resulted in a CI of 24.97% (Figure 1B). The inhibitory effects of pre-harvest MeJA treatments on post-harvest CI were better than those of the post-harvest treatment (Figure 1 and Figure 2A).

Lemon fruits lost weight either in the control or treated fruits during cold storage. The initial weight was reduced by MeJA irrespective of the concentration without significant differences between pre- and post-harvest treatments. At the end of the experiment, the control fruits lost 3.5% of their initial weight, while the fruit treated with MeJA only lost 2.1–2.7% of their initial weight. Pre-harvest treatment with 0.3 mM MeJA maintained low levels of weight loss in the lemon fruit (Figure 2B).

### 3.2. Changes in Texture during Cold Storage

**Figure 3:**
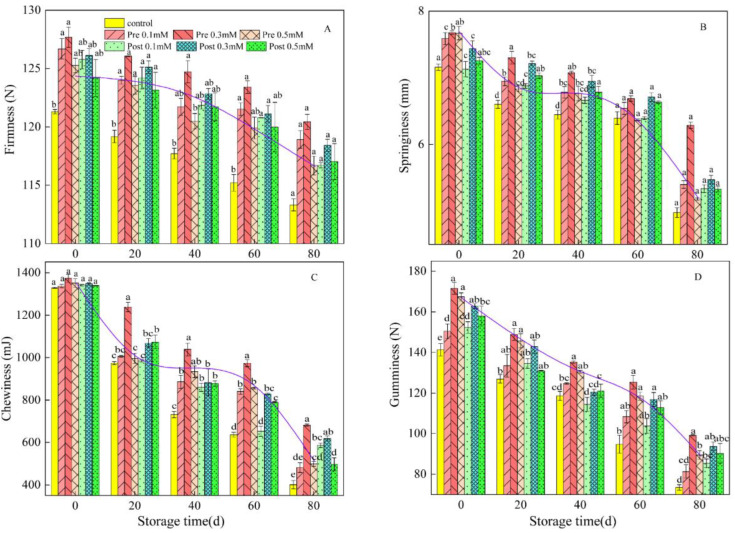
Effect of pre-harvest (Pre) and post-harvest (Post) methyl jasmonate treatments on firmness (**A**), springiness (**B**), gumminess (**C**), and chewiness (**D**) of lemons in storage at 7–10 °C. Data are the mean ± SE of three replicates of five fruit. Different lowercase letters show significant differences among treatments for each sampling date at *p* < 0.05 level.

In this study, firmness, springiness, gumminess, and chewiness were assessed as measures of fruit sensory quality. It should be noted that all the fruits were at the same ripening stage at the beginning of the sensory quality test. As shown in Figure 3, fruit firmness in the control fruit at harvest was 121.39 ± 2.71 N, and it was 113.31 ± 3.18 N at the end of the storage period. Pre-harvest MeJA treatments increased firmness by 3.0 to 5.0% compared with that of the control fruit (Figure 3A). The rate of springiness decline increased with increasing storage time. The springiness of the control fruit at harvest was 7.16 ± 0.03 mm and reached a final level of 4.98 ± 0.27 mm at the last sampling date. The springiness of pre-harvest MeJA-treated lemons ranged from 4.1% to 26.3% (Figure 3B). Fruit chewiness at harvest in control fruit was 1328.23 ± 2.32 mJ, and it was 401.0 ± 5.5 mJ at the end of the storage period. Pre-harvest MeJA treatments increased chewiness by 20.1 to 69.9% compared with control fruit (Figure 3C). Fruit gumminess at harvest in the control fruit was 141.30 ± 3.14 N, and it was 73.54 ± 1.33 N at the end of the storage period. Pre-harvest MeJA treatments increased fruit gumminess by 10.7 to 34.8% compared with that of the control fruit (Figure 3D). Similar results were also found in post-harvest MeJA treatments. However, the pre-harvest MeJA treatments had a greater effect on maintaining fruit sensory quality in comparison to post-harvest MeJA treatments, with the pre-harvest treatment at 0.3 mM MeJA having the greatest effect.

### 3.3. Internal Quality during Cold Storage

**Figure 4:**
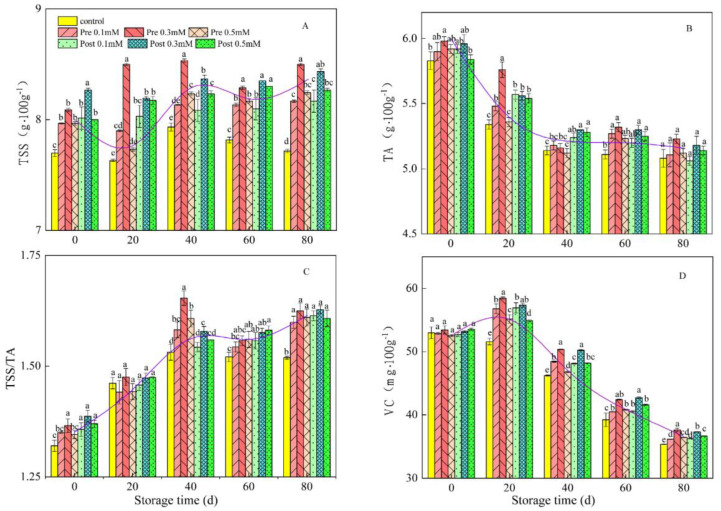
Effect of pre-harvest (Pre) and post-harvest (Post) methyl jasmonate treatments on the TSS (**A**), TA (**B**), TSS/TA ratio (**C**) and VC (**D**) in lemons in storage at 7–10 °C. Data are the mean ± SE of three replicates of five fruit. Different lowercase letters show significant differences among treatments for each sampling date at *p* < 0.05 level.

As shown in Figure 4, pre- and post-harvest MeJA treatment increased the fruit TSS, TA and Vc content during storage, although no significant differences were found between both treatments. Pre-harvest MeJA treatments at 0.3 and 0.5 mM changed the TSS content by 8.50% and 8.24% (Figure 4A), and TA by 5.23% and 5.12%, respectively, compared with that of the control fruit at the end of the storage period (Figure 4B). Similarly, post-harvest MeJA treatments at 0.3 and 0.5 mM changed the TSS content by 8.43% and 8.27% and TA by 5.18% and 5.14%, respectively, compared with that of the control fruit at the end of the storage period. In addition, the TSS/TA ratio was higher (1.63) in fruit treated pre-harvest with 0.3 mM MeJA compared with the ratio in the control fruit (1.54) and fruit from all other treatments. Moreover, TSS and TSS/TA were higher in the pre-harvest MeJA-treated fruit (8.50% and 1.63, respectively) compared with that in the post-harvest MeJA-treated fruit (8.43% and 1.60, respectively) (Figure 4C). The V_C_ content of the control fruit significantly changed from 52.98 mg·100 g^−1^ at harvest to 35.36 mg·100 g^−1^ at the end of the experiment, while that of fruit treated pre-harvest with 0.3 mM MeJA only reduced to 37.64 mg·100 g^−1^. Similarly, post-harvest MeJA treatments at 0.3 mM significantly reduced the decrease in V_C_ content, resulting in a V_C_ content of about 1.06 times that of the control fruit on day 80 (Figure 4D).

### 3.4. Antioxidant Systems and Electrolyte Leakage during Cold Storage

**Figure 5:**
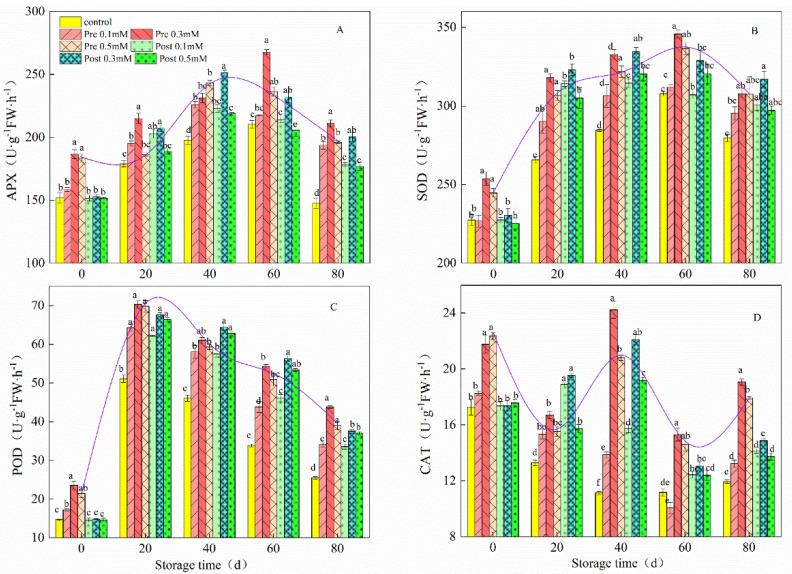
Effect of pre-harvest (Pre) and post-harvest (Post) MeJA treatments on APX (**A**), SOD (**B**), POD (**C**) and CAT (**D**) in lemons in storage at 7–10 °C. Data are the mean ± SE of three replicates of five fruit. Different lowercase letters show significant differences among treatments for each sampling date at *p* < 0.05 level.

As shown in Figure 5A, APX activity was significantly higher in lemon fruit treated pre-harvest with MeJA than in the control fruit (*p* < 0.05). There were significant differences between pre-harvest MeJA-treated lemon fruit and control fruit, APX activity increased slightly over the first 20 d, both in the MeJA-treated and control fruit, then increased sharply from 40 d, followed by a decline. Pre-harvest MeJA treatments at 0.3 mM showed the highest APX activity (267.66 U g^−1^ h^−1^); the highest APX activity in the control was 210.33 U g^−1^ h^−1^. The highest SOD activity (345.74 U g^−1^ h^−1^) was observed in fruit treated pre-harvest with 0.3 mM MeJA at 60 d of storage. Additionally, compared that with control fruit (Figure 4C), POD activity in fruit treated post-harvest with MeJA was higher. POD activity increased sharply, both in the MeJA-treated and control fruit, for the first 20 d, with POD activity reaching 70.33 U g^−1^ h^−1^ in the 0.3 mM MeJA treatment and 51.08 U g^−1^ h^−1^ in the control treatment. After 20 d, POD activity decreased. CAT activity was observably (*p* < 0.05) higher in the fruit treated pre-harvest with MeJA, compared with the control fruit throughout the cold storage period. (Figure 4D).

**Figure 6:**
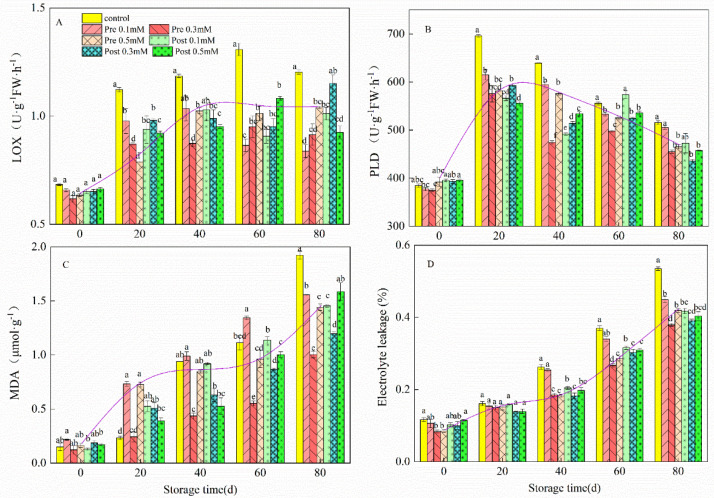
Effect of pre-harvest (Pre) and post-harvest (Post) methyl jasmonate treatments on LOX (**A**), PLD (**B**), MDA (**C**) and electrolyte leakage (**D**) in lemon in storage at 7–10 °C. Data are the mean ± SE of three replicates of five fruit. Different lowercase letters show significant differences among treatments for each sampling date at *p* < 0.05 level.

Pre- and post-harvest MeJa treatment inhibited the LOX, PLD, MDA, and electrolyte leakage (EL) of fruit at harvest and during storage (Figure 6). In this study, compared with the control lemons, LOX (Figure 6A) and PLD (Figure 6B) activities were significantly inhibited (*p* < 0.05) in MeJA-treated lemons. During refrigeration, the MDA content of lemon fruits increased and MeJA treatment significantly inhibited the MDA content of lemon fruits at the end of the storage period (Figure 6C). The MDA content in pre-harvest 0.3 mM MeJA-treated fruit was 47.8% lower than that in the control fruit on day 80. The EL of all the fruits increased rapidly when they were kept in cold storage, but the EL level of the control fruits was higher than that of the fruits treated with MeJA (Figure 6D). At the end of the experiment (80 d), EL in pre-harvest 0.3 mM MeJA-treated fruit was 29.11% lower than that in the control fruit, while that in the post-harvest 0.3 mM MeJA-treated fruit was 25.54% lower than that in the control fruit. Pre-harvest MeJA application was far more effective than post-harvest MeJA application in regard to EL.

### 3.5. PCA Analysis

**Figure 7:**
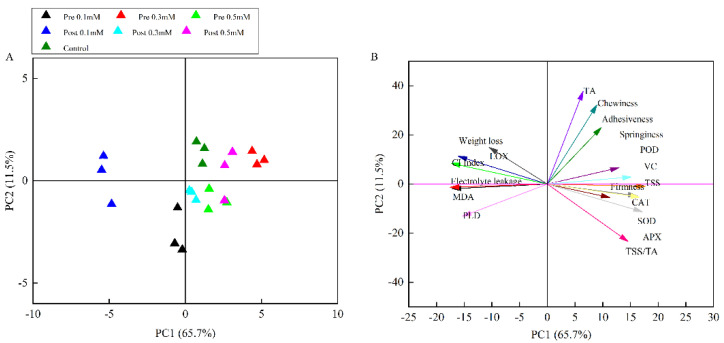
Principle component analysis (PCA) of 17 important indexes measured at the last sampling date of the lemons. (**A**) Score plot of PC2 against PC1; (**B**) Load plot of PC2 against PC1.

PCA was performed according to the correlation matrix designed from measured parameters in different treatments after 80 days of cold storage (Figure 7A,B). Figure 7A represents the first two principal components (PC1 and PC2), which covered 77.2% of the total variance. PC1 was calculated for the maximum amount of total variance (65.7%) while PC2 accounted for 11.5%. The treatments tested were almost completely separated from each other (Figure 7A). Furthermore, there was an obvious relationship between the CI, fruit quality, antioxidant activity, and membrane lipid hydrolysis-related enzymes (Figure 7B). Acute angles were found between the vector arrows of CI, WL, LOX, MDA, PLD and EL, and this indicates that CI is positively correlated with WL, LOX, MDA, PLD and El. However, there was an obtuse angle between the CI and the vector arrows of fruit texture, and between the vector arrows of TSS, TA, and antioxidant activity, which indicates that CI is negatively correlated with fruit texture, TSS, TA, and antioxidant activity. 

## 4. Discussion

Eureka lemons, like other citrus fruit, are susceptible to physiological, biochemical and pathological disorders leading to quality losses during storage and transportation [23]. Cold storage is probably the most effective method for maintaining fruit quality and prolonging the post-harvest life the of fruit, however, fruit of many citrus cultivars are sensitive to developing CI of the peel, which significant downgrades external fruit quality [5]. Pre-harvest environmental conditions may have a strong effect on post-harvest chilling responses in cold-stored citrus fruit [24]. The results of this study suggest that pre-harvest application of MeJA had a considerable effect on reducing CI compared with the post-harvest application of MeJA at the same concentration. The positive effect of pre- or post-application of MeJA on reducing CI in lemon fruit has been previously reported and is thought to be linked to enhanced antioxidant defense mechanisms [25]. Reduced weight loss has been shown for post-harvest applications of MeJa in ‘Arrayana’ mandarins [26]. This is supported by the results from this study which suggest that the control lemon fruit exhibited obvious CI symptoms after cold storage over 40 days at 7–10 °C, however, treatments with pre- or post-application of MeJA at three concentrations (0.1, 0.3, and 0.5 mM) alleviated the CI symptoms and WL of ‘Eureka’ lemons, with the most effective doses being pre MeJA at the concentration of 0.3 mM (Figure 1 and Figure 2). Notably, when the concentration of MeJA increased to 0.5 mM, CI increased, as well as increases in WL, indicating that excessive MeJA concentrations release negative impacts on CI [27]. WL is mainly caused by transpiration through fruit peel [28]. Furthermore, WL is caused by metabolic activities, such as respiration, and cellular breakdown due to CI damages at low temperatures [29]. Thus, the reduction of WL found in treated lemon fruit could be attributed to their effect on maintaining cell integrity at low temperatures according to previous report on oranges [30].

Visual appearance, freshness and firmness are key factors that define the external quality of lemon [31]. It has been reported that prolonged storage time leads to progressive degradation, resulting in a decrease in the overall sensory acceptability of fruit [32]. Texture measurement system analysis has high sensitivity and objectivity and has been applied to plums [20], dates [33], and apples [34]. In our experiment, four representative indicators including firmness, springiness, gumminess, and chewiness were obtained using TPA, and our results are consistent with the earlier report of Yang et al. [35], who found that prolonged storage time would cause decreased springiness, hardness, viscosity, chewiness and viscosity of fruit (Figure 1). Brummell et al. (2001) [36] showed that firmness provides information about the rate of softening and the storage capacity of fruit, previous studies on jackfruit have shown that the changes of fruit firmness are closely related to pectic acids, hemicelluloses, celluloses, cell expansibility, cell cohesion and assimilation, etc. [37,38]. The post-harvest applications of MeJA maintained higher firmness in ‘Kinnow’ mandarin [39]. Our study showed that when the concentration of MeJA was the same, the firmness of lemons with pre-harvest MeJA treatment was higher than that of post-harvest MeJA treatment, and pre-harvest treatment at 0.3 mM MeJA presented the highest values amongst all treatments, but no significant differences were detected among them. The other three properties, i.e., springiness, gumminess, and chewiness, were similar to the firmness in different treatments during cold storage.

Cold storage periods have been reported to influence the TSS, TA, V_C_ and total antioxidant capacity in lemon fruits and other fruits [28,40]. TSS is a basic osmotic regulator for cold acclimation of higher plant cells [41]. In our study, the TSS in lemon juice exhibited a declining trend from 0 to 20 days of cold storage and increased with the extension of cold storage periods (Figure 4). An increase in TSS with the extension of storage periods may be attributed to numerous catabolic processes during fruit storage periods [3]. A previous study has shown that the increase in TSS in lemon fruits during storage may be caused by water loss [42]. We found a similar relationship was observed between mean weight loss and mean TSS (Figure 2 and Figure 4). In addition, pre-harvest application of MeJA had a considerable effect on increasing TSS content compared with post-harvest application of MeJA at the same concentration, the effect of pre-treatments on enhancing TSS could be due to an increase in the net photosynthetic rate of tree, which would lead to increased sugar accumulation [28]. The decreasing trend in the mean TA was observed during cold storage periods (Figure 4). Serna-Escolano et al. (2021) [28] suggested that TA decreased significantly in all treatments during cold storage, and pre-harvest treatments with MeJA and SA lead to TA values which are significantly higher in treated fruit than in the control. Similar findings of the decreased TA with the extension of storage periods were observed in our study. V_C_ serves as a main antioxidant which can directly scavenge ROS. V_C_ levels in plants can be regulated by the synergistic action of various related enzymes [43]. The higher V_C_ content may indicate a superior antioxidant capacity in MeJA-treated lemons [44]. Our data showed that the mean V_C_ content in the juice of cold stored lemon fruit was not significantly affected by the pre- and post-harvest treatment with MeJA (Figure 4).

Damage to plant membrane structures caused by CI is common in cold storage [45]. Membrane lipid hydrolysis-related enzymes, such as PLD and LOX, conduce the degradation of membrane lipids, which contributes to a loss of cell membrane integrity [46], which could be responsible for the brown spots in lemon surfaces. In this study, PLD and LOX activities were higher at 80 d compared with at 0 d in all treatments. These findings indicate that the increased activities of these enzymes correlated with the decreasing storability of lemons. These results are consistent with previous reports that the increased activities of membrane lipids hydrolyzing-related enzymes were not conducive to the storage of pears [47], and longans [48]. MDA and EL have been commonly regarded as physiological markers of a loss of membrane integrity and lipid membrane peroxidation caused by chilling stress. They are usually used to assess membrane damage related to CI severity [44]. Damage or deterioration of plant membranes can lead to a significant increase in EL [49]. In the current study, low temperature treatment resulted in the accumulation of MDA in the lemon fruit, while MeJA treatment observably inhibited the increase in MDA content. We report herein that both pre- and post-harvest MeJA treatment can alleviate the symptoms and severity of CI, maintain membrane integrity, and reduce EL. Pre-harvest MeJA application was found to be far more effective than post-harvest application, with pre-harvest treatment at 0.3 mM MeJA having the greatest effect.

Maintaining a high ROS scavenging capacity and low activities of membrane lipid-degrading enzymes contribute to maintaining the cell membrane function and, therefore, enhance the protection of post-harvest fruit [50]. Previous studies have shown that MeJA can mediate intra- and inter-plant communication, coordinating plant defense responses, including antioxidant systems [50]. Serna-Escolano et al. (2019) [18] showed that MeJA treatment during fruit development led to an increase in the activities of CAT, POD and APX enzymes in the juice of ‘Verna’ and ‘fino’ lemon varieties at harvest. In this study, the antioxidant activity of the APX, SOD, CAT, and POD enzymes increased during cold storage in all the samples, and the increase in the fruit treated with both pre- and post-harvest MeJA was significantly higher than in the control fruit. APX, SOD, CAT, and POD are enzymes which play important roles in H_2_O_2_ decomposition and have a very specific function in detoxifying ROS [51]. In our study, according to PCA, WL, LOX, MDA, PLD, EL were considered as related parameters to CI damages. These parameters were apparently associated with control samples in which the severity of CI reached the maximum. SOD, POD, CAT and APX activities as well as fruit texture were apparently associated with MeJA treatments. Thus, it could be concluded that enhancing the chilling tolerance of fruit was related to enhancement of the antioxidant system and osmoregulation.

## 5. Conclusions

In summary the application of MeJA before harvest was much more effective than the application of the same concentration of MeJA after harvest. Pre-harvest application of MeJA (0.03 mM) extended the cold storage (7–10 °C; 90–95% RH) life of ‘Eureka’ lemon fruits up to 80 d by reducing CI, weight loss, firmness loss, increasing the activities of antioxidant enzymes, and maintaining higher levels of TSS, TA and sensory attributes.

## Figures and Tables

**Table 1:** Parameter definition and calculation of puncture testing and texture profile analysis.

Parameters	Definition	Units
Firmness	Maximum value of the peak force of the resulting curve	N
Cohesiveness	The ratio of the positive peak area of the second extrusion cycle to the positive peak area of the first extrusion cycle	Ratio
Springiness	The ratio of the height of the second compression to that of the first compression	mm
Gumminess	Firmness × cohesiveness	N
Chewiness	Firmness × cohesiveness × springiness	mj

Note: The texture parameters in the table are defined by reference to Qiu et al. (2021) and TMS-PRO operating guidelines.

## Data Availability

All data generated or analyzed during this study are included in this published article.
